# Sir Francis Bacon’s Cipher Story

**Published:** 1894-06

**Authors:** E. K. Tullidge


### BOOK NOTICES.

SIR FRANCIS BACON'S CIPHER STORY. Discovered and Deciphered by Orville W. Owen, M. D. Vol. I. Detroit and New York. Howard Publishing Co. 1893. Paper cover, price, 50 cents.

A WORD FOR SHAKESPEARE. *Palmam qui meruit ferat.* A few men, whose opinion goes for something in this world, have allowed themselves to be put on record as believing that Francis Bacon wrote the plays attributed to William Shakespeare. Others are not so sure that Bacon wrote the plays as they are that the man Shakespeare did not and could not have written them. It does not, therefore, seem altogether an idle thing to strive to show the utter absurdity involved in supposing that any one but Shakespeare could have written the plays attributed to him.

Let us, for the present, leave Bacon entirely out of the question. No need of Bacon, if we can prove it to be absurd to imagine that any one but Shakespeare could have written the plays. Granting, for the sake of argument, that Shakespeare did not write the plays, what then must have happened? Something like the following:

Some young man of unusual ability feels driven to give expression to the multitudinous thoughts that are swarming in his brain. He has the poet's eye in fine frenzy rolling, glancing from heaven to earth, from earth to heaven. As his imagination bodies forth the forms of things unknown, his pen can turn them to shapes, and give to airy nothing a local habitation and a name. But totally unlike other young men of this description, he cannot bear the idea of being known as the author of his own effusions. There are difficulties in the way of writing anonymously. Much the best plan seems to him to be to seek out some one who will consent to father his productions, to be the stalking-horse, under cover of which he can, with safety, discharge his wit and other shafts of his genius, without the danger of being discovered. The promptings of his genius urge him to write for the stage. So he hies him to the Blackfriars' Theatre to find some one suitable for his purpose among "The Lord Chamberlain's Servants." His quick eye selects one of the players, who, it seems to him, possesses the most plentiful lack of wit and want of self-respect necessary for the undertaking.

It was one William Shakespeare, lately come up from Stratford-on-Avon, to try his fortune in the great world of London. Somehow, the very fact of his being one of the players in this leading theatrical company, hardly seems to warrant such a remarkable want of brain as some seem to wish us to believe of this man. To be an actor in the leading theatrical company of London for twenty-five years, does not seem to be altogether consistent with the idea of his being such a very simple-minded man, such an out and out blockhead. But this only as a hint. Let us proceed. The Great Unknown, so anxious to give utterance to his seething thoughts, yet no aspirant for fame, but rather amorous of oblivion, seeks an interview with the young man from Warwickshire, and acquaints him with the great favor he has to ask of him. The wondrous power, which we know the author of the plays possessed, of reading the human heart, did not mislead him in this instance. The youthful Shakespeare lends a willing ear and gives consent, little dreaming then of the golden harvests he was to reap thereby, and of the way in which his name was to fill the trump of future fame. Arrangements having thus been happily concluded, there is nothing left but to begin. And first he wisely tries his hand at tinkering up some old plays. The three parts of King Henry VI seem manifestly to have been the first productions of his pen. Strange to say, these plays were very clearly written by more than one person. The Second and Third Parts were first published in 1594 and 1595 respectively, with a different title from that in which they appear in the folio of 1623, and with a very different text. In their earlier form, the workmanship of three hands is plainly seen by the most competent critics, those, namely, of Shakespeare himself (as they ignorantly suppose), Marlowe, and Greene. This was exactly in accordance with the custom of the day for one, two, three, or even four persons to write a play together. How did Shakespeare manage to deceive those men by making them think that he was writing in partnership with them, when in reality he was doing nothing of the kind, being in fact utterly incapable of it? This has always seemed to the writer to be an absolutely fatal objection to the idea that the plays attributed to Shakespeare were written by anybody but Shakespeare himself. For it is beyond any possibility of intelligent questioning that others of the plays were written in partnership, notably "The Two Noble Kinsmen" and "King Henry VIII." When "The Two Noble Kinsmen" was first published, it was announced on the title-page "Written by the memorable worthies of their time, Mr. John Fletcher and Mr. William Shakespeare, Gentlemen." The most searching investigation of the internal evidence proves this conclusively. Some of the work is far beyond the capacity of any man but the author of the plays attributed to Shakespeare. The rest of the work is exactly in the style of Fletcher. Internal evidence proves the same of Henry VIII. Joint authorship of plays, as already noted, was one of the most common things of the day, and naturally the man Shakespeare would be called upon to write in partnership with some of the best dramatists living. According to the theory of those who insist that he did not write the plays attributed to him, he could not possibly have written in partnership with these men. And yet they thought that he had done so. How was he able to deceive them? He must have had a wonderful genius for deceit if for nothing else. The plan of proceeding must of course have been for Shakespeare to be in constant and intimate communication with the Great Unknown, so that he might be provided with the parts of the play expected from him. And yet it is hard to see how he could have gotten along, even so, without exciting suspicion. How was it possible for Shakespeare to apparently write a play in partnership with Fletcher, and avoid personal interviews and interchange of opinion with him as the best way for the working out of the plot, etc.? But being such an ignoramus, as the Baconians would make him out, this was impossible for him to do.

It did not take long for the Great Unknown to prove his superiority, and the man Shakespeare reaped the substantial benefits in reputation and a well-filled purse. Very interesting testimony has come down to us to that effect. The play-writer, Robert Greene, in consequence of his profligate life, was reduced to beggary, and died miserably in the house of a poor shoemaker, in 1592. In his tract called "A Gratsworth of Wit," published after his death, he makes the following unmistakable and spiteful fling at Shakespeare: "Yes, trust them not; for there is an upstart crow beautified with our feathers, that, with his 'tiger's heart, wrapped in a player's hide,' supposes he is as well able to bombast out a blank verse as the best of you; and, being an absolute Johannes Fac-totum, is in his own conceit the only Shake-scene in a country." The quotation "tiger's heart wrapped in a player's hide," is a parody on a line from the Third Part of King Henry VI, i. 4, player being substituted for woman.

The phrase "bombast out a blank verse" must refer to Shakespeare as an author for the following reason: Marlowe was the first to substitute blank verse for rhyme. In this the author of the plays attributed to Shakespeare followed him, so that Greene is here intimating to Marlowe that Shakespeare is trying to outrival him in his own invention. Marlowe and Shakespeare seem, both of them, to have been offended with the liberties thus taken with them. For before the end of the same year, Chettle, who had brought out the first tract, published another, entitled, *Kind Heart's Dream*, and endeavors to make amends in the following words: "With neither of them that take offense was I acquainted, and with one of them (Marlowe) I care not if I never be; the other I did not so much spare as since I wish I had, because myself have seen his demeanour no less civil than he excellent in the quality he professes: besides divers of worship have reported his uprightness of dealing, which argues his honesty, and his facetious grace in writing that approves his art." The plan is working well. The real author and the supposed author are both satisfied. Still more substantial rewards are in the near future.

In the year 1593 was published the poem, "Venus and Adonis," and dedicated to the Earl of Southampton; and, in the year following, "Lucrece" was published and dedicated to the same nobleman. The "Venus and Adonis" immediately achieved a great popularity. Meres, in his *Wit's Treasury*, 1598, speaks of it thus: "As the soul of Euphorbus was thought to live in Pythagoras, so the sweet, witty soul of Ovid lives in mellifluous and honey-tongued Shakespeare: witness his 'Venus and Adonis,' his 'Lucrece,' his sugared sonnets among his private friends." There is very strong evidence that the man Shakespeare was the recipient of a most munificent gift from the Earl of Southampton, in return for the dedication of the poems to him. It is said that he gave Shakespeare no less than £1,000, equal to about $30,000 in the money of our time, to enable him to effect a purchase which he knew him to be desirous of making, probably the Globe Theatre. Surely, this man Shakespeare must have been a master actor off the stage, if not on it, in order to support his growing reputation without anybody suspecting that he was not really entitled to it. But so it goes on. Play succeeds play, the most wonderful masterpieces of human genius, and no one suspects that they are not by their reputed author! And consider more fully what this means.

The plays attributed to Shakespeare were certainly written by a man of irrepressible wit and humor. What might naturally be expected from the conversation of a man who created the character of Mercutio and Falstaff? Of course, if Shakespeare did not write the plays, he was not capable of any such conversation as would naturally be expected of their author. And yet it never occurs to any of his fellow-actors to say, "How comes it to pass that Will Shakespeare can make his characters say such bright and witty things, and yet be such a dull fellow when it comes to using his tongue?" Remember, too, what a jovial set these playwriters and actors were. Shirley, for instance, writes thus to Ben Jonson:

"What things have we seen
Done at the Mermaid! heard words that have been
So nimble, and so full of subtile flame,
As if that every one from whence they came
Had meant to put his whole life in a jest,
And had resolved to live a fool the rest
Of his dull life."

Of this merry crowd Shakespeare would, of course, be expected to be the very first and foremost. But if the Baconian theory be true, he could not have had a word to throw away on a dog on such occasions. And yet, somehow or other, he did gain the reputation of carrying off the palm as a conversationalist as well as a writer.

Dr. Thomas Fuller, who could easily have conversed with men who were present on such occasions, thus writes of him: "Many were the wit-combats betwixt him and Ben Jonson; which two I behold like a Spanish great galleon and an English man-of-war. Master Jonson, like the former, was built far higher in learning; solid, but slow, in his performances; Shakespeare, with the English man-of-war, lesser in bulk, but lighter in sailing, could turn with all tides, tack about, and take advantage of all winds, by the quickness of his wit and invention." If Shakespeare's detractors could only see their way clear to maintaining that the real author of the plays had somehow got possession of the ring of Gyges, which made its wearer invisible, their theory would be rendered infinitely more plausible than it now is. This would have put it into the power of the Great Unknown to be at Shakespeare's elbow at such trying times and tell him what to say.

But as great, if not even greater difficulties than this, present themselves to the mind. How was it possible for Shakespeare to avoid conversation with some of the keenest witted men of his day on all topics connected with his art which of course had its bearing upon all departments of human life? Men would expect him to be in conversation exactly what they thought his own pen had so beautifully depicted in the following lines from "A Lover's Complaint":

"So on the tip of his subduing tongue
All kind of argument and question deep,
All replication prompt, and reason strong,
For his advantage still did wake and sleep;
To make the weeper laugh, the laugher weep,
He had the dialect and different skill,
Catching all passion in his craft of will."

But who more incapable of using his tongue in such fashion as this than Shakespeare, if he was what his detractors wish us to believe he was! And yet we know what were the personal impressions made by him upon one of the literary giants of the day. Ben Jonson was intimately acquainted with him, and the poem he wrote in memory of Shakespeare is the most glowing eulogy ever penned, enough of itself to confer immortality on the writer and on the subject of it. Pray note the words with which he introduces it to the reader. "To the Memory of My Beloved, the Author, Mr. William Shakespeare." We quote a line or two to show the estimate he put upon the plays:

"Soul of the age,
Th' applause, delight, the wonder of our stage,
My Shakespeare rise!

* * * * * when thy socks were on,
Leave thee alone for the comparison
Of all that insolent Greece or haughty Rome
Sent forth, or since did from their ashes come.
Triumph, my Britain! thou hast one to show,
To whom all scenes of Europe homage owe.
He was not of an age, but for all time;
And all the Muses still were in their prime,
When, like Apollo, he came forth to warm
Our ears, or like a Mercury to charm.
Nature herself was proud of his designs,
And joyed to wear the dressing of his lines;
Which were so richly spun, and woven so fit,
As since she will vouchsafe no other wit:
The merry Greek, tart Aristophanes,
Neat Terence, witty Plautus, now not please;
But antiquated and deserted lie,
As they were not of Nature's family.

Look how the father's face
Lives is his issue; even so the race
Of Shakespeare's mind and manners brightly shines
In his well-turned and true-filed lines."

Many years after Shakespeare's death Ben Jonson was charged with malevolence toward him, which charge he repelled in the following words: "I loved the man, and do honor his memory, on this side idolatry, as much as any. He was indeed honest, and of an open and free nature; had an excellent fantasy, brave notions, and gentle expressions.'' There is, then, this fact before us. Ben Jonson fully realized the transcendent excellence of the plays attributed to Shakespeare. He was also intimately acquainted with the man Shakespeare, and the personal impression which Shakespeare made upon him was exactly what it was to be expected the real author of the plays would make. He made the same impression upon his fellow-actors. When John Heminge and Henry Condell brought out the first complete edition of his plays, in the folio of 1623, they declared that they had done so "without ambition either of self-profit or fame; only to keep the memory of so worthy a fellow alive as was our Shakespeare." Behold, then, what some would have us to believe: that a man, almost an ignoramus, could for more than twenty-five years sustain the reputation of having written some of the masterpieces of human genius without any of his most intimate associates suspecting his incapacity! The personal impression was fully equal, if not more than equal, to the literary impression. It would require as great a genius to support such a character successfully as to write the plays.

And the Great Unknown was Bacon! As well make the absurdity as towering as possible while we are about it. Bacon's Essays are great; but they are mere child's play compared with what the author of the plays was capable of. And yet it is clear that Bacon has expended the full force of his mind upon the Essays, improving upon them and enlarging them as the years went on. What did the man who wrote the Essay on Love, know about love as it is depicted in the plays? The man who wrote that essay betrays an utter want of sympathy with the all-devouring passion. He knew as much about love, in the way that the author of the plays knew of it, as the average American knows about Chinese.

A new aspirant for fame, and possibly something more substantial, is clamoring for a hearing on the marvelous discovery he claims to have made. Bacon, according to him, was the author not only of the plays attributed to Shakespeare, but also of the plays attributed to Robert Greene, George Peel, and Christopher Marlowe; the author also of all the works of Edmund Spenser and of *The Anatomy of Melancholy* of Robert Burton! In short, "Bacon was the whole Elizabethan age." And all these works were written about the year 1623, years after the reputed authors were in their graves! None of these plays were ever acted, but were all written in the course of a year or so in order to weave into them a secret history of the most blood-curdling description! And this in the face of the Dedication and Address prefixed to the Folio of 1623 by Shakespeare's fellow-actors! The title of the book is *Sir Francis Bacon's Cipher Story*. Professor Max Muller tells us that "Cipher is the Arabic *cipron*, which means 'empty,' a translation of the Sanskrit name of the 'nought.'" It is well to go back to the original derivation of words sometimes. E. K. TULLIDGE.

